# Editorial: Diagnosis and therapy pediatric hematological malignancies—recent progress—volume II

**DOI:** 10.3389/fped.2025.1749385

**Published:** 2025-12-04

**Authors:** Joanna Zawitkowska, Monika Lejman, Katarzyna Derwich

**Affiliations:** 1 Department of Pediatric Hematology, Oncology and Transplantology, Medical University of Lublin, Lublin, Poland; 2 Independent Laboratory of Genetics Diagnostics, Medical University of Lublin, Lublin, Poland; 3 Department of Pediatric Oncology, Hematology and Transplantology, Poznan University of Medical Sciences, Poznan, Poland

**Keywords:** hematological malignancies, children, diagnosis, therapy, target theapy

Pediatric hematological malignancies represent a dynamic and evolving field in oncology, where continuous progress in diagnostics and therapy has transformed patient outcomes. Over recent decades, survival rates have improved dramatically, largely due to the integration of precision medicine and risk-adapted protocols (1, 2). The introduction of minimal residual disease (MRD) monitoring has become a cornerstone for tailoring treatment intensity and predicting relapse risk (3). Despite these achievements, the clinical landscape remains complex. Treatment-related toxicity, refractory disease, and relapse continue to pose significant challenges (4). Conventional chemotherapy intensification has reached its limits, prompting a paradigm shift toward targeted and immune-based strategies. Novel agents directed at specific genetic lesions, monoclonal antibodies, and cellular therapies such as CAR-T cells are redefining therapeutic possibilities for children with high-risk or relapsed disease (5–8).

This special issue brings together cutting-edge research and expert perspectives on diagnostic innovations, therapeutic advances, and future directions in pediatric hematological malignancies. Our goal is to provide clinicians and researchers with insights that will guide the next generation of personalized care.

Xue et al. documented two pediatric cases of systemic mastocytosis (SM) coexisting with AML1::ETO-positive acute myeloid leukemia (AML), driven by previously unreported KIT exon 11 mutations. Both patients underwent allogeneic hematopoietic stem cell transplantation but later developed refractory disease, resistant to multiple salvage regimens. Remarkably, avapritinib therapy produced exceptional clinical responses in these complex scenarios [Xue et al.]. Zhang et al. investigated the clinical features and prognostic significance of gene mutations in pediatric T-cell acute lymphoblastic leukemia (T-ALL). The most frequent alterations were NOTCH1 (58.3%), FBXW7 (19.4%), and PTEN (17.4%). Of 1,262 mutations identified, 50 had a frequency above 1%. Common mutations were not associated with 5-year overall survival; however, patients with higher NOTCH1 mutation burden had lower day-15 MRD ≥0.01% and better survival outcomes. This study emphasizes the role of next-generation sequencing (NGS) in molecular classification, risk stratification, and prognosis, and highlights the clinical relevance of NOTCH1 variant allele frequency in predicting treatment response [Zhang et al.]. Alfaro-Hernández et al. assessed the impact of standardized immunophenotyping and centralized MRD monitoring on early mortality in children with B-ALL treated in public hospitals in southern Mexico. Their findings show that MRD-informed response assessment significantly reduced early mortality and improved one-year survival in socioeconomically vulnerable populations, demonstrating the feasibility and value of centralized diagnostic platforms in low- and middle-income countries (LMICs) [Alfaro-Hernández et al.]. Hassan et al. analyzed the incidence and risk factors of asparaginase-associated pancreatitis (AAP) in pediatric ALL patients. Older age and high-dose asparaginase were independent risk factors. The authors stress that re-challenging asparaginase requires careful consideration of the risk of recurrent pancreatitis vs. relapse risk [Hassan et al.]. Budair et al. reported a rare presentation of pediatric chronic myeloid leukemia: priapism. This condition demands urgent medical and surgical intervention to prevent long-term complications such as erectile dysfunction [Budair et al.]. Pelegrina et al. conducted a retrospective study of 310 pediatric patients undergoing hematopoietic cell transplantation (HCT) for acute leukemias in Brazil. Results highlight increased relapse risk associated with advanced disease status, positive pre-HCT MRD, and mixed donor chimerism post-transplant [Pelegrina et al.]. Jenabzadeh et al. described the first reported case of Rosai-Dorfman disease coexisting with high-risk pre-B-cell ALL in an adolescent receiving chemotherapy. Unfortunately, the patient relapsed and died due to fungal infection [Jenabzadeh et al.]. Cardoso de Castro et al. systematically reviewed rituximab use in children and adolescents with high-grade mature B-NHL and performed a meta-analysis. Rituximab improved outcomes in first-line therapy and overall survival in relapsed/refractory cases. However, access in Brazilian public hospitals remains limited and dependent on institutional resources [Cardoso de Castro et al.].

## Summary

Recent studies underscore the complexity and diversity of pediatric hematological malignancies. Advances in molecular profiling, MRD-guided therapy, and targeted treatments such as avapritinib and rituximab are reshaping clinical practice. However, challenges persist, particularly in LMICs, where access to standardized diagnostics and novel therapies remains limited. These findings highlight the need for global collaboration to ensure equitable implementation of precision medicine and improve survival outcomes for all children.
